# Does duration on antiretroviral therapy determine health-related quality of life in people living with HIV? A cross-sectional study in a regional referral hospital in Kenya

**DOI:** 10.3402/gha.v7.23554

**Published:** 2014-04-07

**Authors:** Edwin Mûnene, Björn Ekman

**Affiliations:** 1 Nyeri Provincial General Hospital, Nyeri, Kenya; 2 Social Medicine and Global Health, Lund University, Malmö, Sweden

**Keywords:** health-related quality of life, HIV, AIDS, Kenya, SF-36, ART

## Abstract

**Objective:**

To measure the extent to which health-related quality of life (HRQoL) in people living with HIV is associated with duration of antiretroviral therapy (ART) after controlling for sociodemographic, clinical, and other therapy-related factors.

**Design:**

Cross-sectional analysis.

**Methods:**

A gender-stratified random sample of 421 participants aged 18–64 years was selected from the patients on ART at a health facility in Kenya. Three hundred and ninety two patients participated in the study, representing a 93% response rate. Data on general physical and mental health functioning status were collected using the SF-36 health survey questionnaire. Hierarchical logistic regression analysis was used to predict the SF-36 summary scores.

**Results:**

In regression analyses, the duration of ART was negatively associated with HRQoL (odds ratio (OR): 0.6, 95% confidence interval (CI): 0.45–0.92) after controlling for sociodemographic, clinical, and other therapy-related factors. Patients with chronic diseases or clinical symptoms of acute illness had significantly worse HRQoL (OR: 0.5, 95% CI: 0.30–0.79 and OR: 0.3, 95% CI: 0.16–0.59, respectively). Therapy interruptions, adverse drug reactions, and World Health Organization stage at initiation of therapy were not associated with HRQoL.

**Conclusion:**

Patients on ART for a relatively longer duration reported poorer HRQoL at the study facility independent of the effect of other therapy-related, clinical, and sociodemographic factors. Program managers and clinicians in the Kenyan health system may need to refocus attention on this subgroup to avert ‘loss to treatment’ that may have negative repercussions on the substantial gains made against the HIV scourge.

While the HIV epidemic remains a major global public health concern, and more so in developing countries, considerable gains have been made in stemming its economic and social toll on communities (1). Antiretroviral therapy (ART) regimens have been a key component to much of these gains as they have contributed to increased longevity and reversal of disease progression (2, 3). However, they are not without challenges as they involve a strict treatment protocol, including adherence to the therapies; the need to handle the adverse drug reactions associated with the potent medicines; and the development of strategies to deal with the psychosocial and other life events associated with living with HIV (4). Although much has been studied on how these and other factors interact to affect patients’ perception of their general health status, more needs to be presented on how these vary over the course of treatment and their joint temporal impact on patients’ self-assessed general health.

Health-related quality of life (HRQoL) is a multidimensional psychometric construct that is distinct from the concept of ‘quality of life’ in that it focuses on those aspects of the human experience and a person's assessment of the same that are amenable to health interventions (5). It derives summary measures from people's subjective evaluations of their physical, mental, and emotional health and functioning. Several self-administered generic and disease-specific instruments have been developed and used in measuring HRQoL in people living with HIV (PLHIV) (6, 7). Most studies assessing HRQoL in PLHIV have been limited largely to the assessment of clinical, psychosocial, and/or sociodemographic HRQoL correlates, with few studies performed in resource-poor settings (8).

Of those that have assessed the variation of HRQoL in PLHIV with time, findings have been mixed. On one hand, in a longitudinal study assessing factors associated with HRQoL, Jelsma et al. (9) found that HRQoL improved in a South African cohort over a 12-month follow-up, although the sample consisted largely of members with similar pretreatment clinical health status. On the other hand, Carrieri et al. (10), in a heterogeneous sample with respect to pretherapy health status, reported general improvements in HRQoL over a similar period in patients with a poorer baseline clinical status but not in those with a moderate-to-normal baseline clinical status. A plausible explanation provided was that those patients initiating therapy when moderately immunocompromised were in the long run more likely to regard the cons of HIV treatment higher than its benefits. Comparable findings have been made in other similarly designed studies (3, 11) as well as qualitative studies (12), where HRQoL has been shown to have differential improvement over time with respect to some other clinical or biodemographic factor(s). Few studies have considered to what extent the HRQoL changes in PLHIV can be independently explained by duration on therapy as the primary predictor or by taking longitudinal measurements past the 12-month mark. This is in light of evidence that biochemical changes attributable to HIV and AIDS, even with treatment, span a wider temporal window (13) and may, through biophysical manifestations, influence patients’ evaluation of their health condition.

The aim of the this study was therefore to investigate to what extent duration on ART in PLHIV predicts patients’ self-perceived health status independent of their current and pretreatment clinical health status as well as other well-known therapy-related and sociodemographic factors associated with HRQoL. If duration of ART is associated with changes in HRQoL, then a good level of understanding of the underlying mechanisms may aid health system and HIV program managers to supplement objective clinical status measurements with self-assessed general health in ensuring an optimal health outcome environment among patients at different stages of treatment.

## Methods

### Design

The cross-sectional study was conducted between February and April 2013 at Nyeri Provincial General Hospital (Nyeri PGH), Nyeri County, Kenya. Nyeri County is located in the central highlands of Kenya, about 150 km north of the capital Nairobi. Nyeri PGH is the main referral hospital in the region, with the catchment area extending to at least four other neighboring counties. As a regional referral hospital, it serves both curative and rehabilitative functions in addition to preventive services to a population of about 700,000. HIV prevalence in Nyeri County is estimated at 4.4% compared to the national average of 6.2% (14).

The study was conducted between February and April 2013. The study's sampling frame was obtained from the list of patients actively taking ART at the hospital as of January 2013, that is, all patients who were enrolled in the ART program in January 2013 or earlier and were still active on treatment. Patients were eligible for inclusion if they were aged between 18 and 64, had been on highly active ART (HAART) for at least 1 month, and had a clinic appointment within the study period. Consequently, from a total population of 1,693 patients on HAART, 1,189 met the inclusion criteria. Since HIV prevalence is biased on gender (1) (and the sampling frame comprised 69.0% females), a final sample of 421 patients was aggregated from a proportionate simple random sample from each of the two gender-stratified groups of the sampling frame. Sample size was determined from the World Health Organization's (WHO) *Practical Manual for Sample Size Determination in Health Studies* (15), where a minimum sample size of 380 would have been adequate to estimate an odds ratio (OR) of at least 1.5 at the 5% significance level.

### Data collection and ethical considerations

Data were collected using paper questionnaires administered in privacy to the patients. Written approval for the study was sought and obtained from the hospital management prior to commencement. All participants were informed of the study's purpose prior to participation, and informed verbal consent was subsequently obtained. They were informed that the services they receive at the hospital would continue as usual whether or not they elected to participate. For those unable to complete the questionnaire for physical, literacy, or other reasons, the questions were read to them as written and in their preferred language (66.1% preferred the Kiswahili to the English version).

### Outcome variables

The 36-Item Short-Form Health Survey (SF-36) (16) instrument was used to collect information on patients’ HRQoL profile. The SF-36 scores health on eight subscales, ranging from physical functioning to mental health, and two summary measures: a physical component score (PCS) and mental component score (MCS) (7, 16). For categorical analyses, PCS and MCS were dichotomized at their respective medians, yielding ordinal binary ‘high’ and ‘low’ variables. To simplify interpretation, a third variable was generated as a Cartesian product of the PCS and MCS, and was dichotomized into ‘high’ and ‘low’ levels so that a participant classified as ‘high’ in the aggregated PCS and MCS variable was also classified as ‘high’ in both the PCS and MCS variables.

### Independent variables

The study's primary explanatory variable (duration of ART) and control variables were obtained from an addendum to the SF-36, clinical, and pharmacy records. The electronic clinical and pharmacy records were imported and collated into a software application with a relational database management system at the backend. The variables were classified into three broad categories:

#### Sociodemographic factors

Information on age and gender was obtained from the pharmacy records, whereas information about the participants’ education, income, marital status, religion, and occupation was captured through the addendum to the SF-36. Table 1 summarizes the variable levels used in analysis.

**Table 1:** Participants’ characteristics and contrast between those on ART for less than 4.5^a^ years versus those on ART for 4.5 years or more

			Duration on ART		
				
		*n* (%^b^)	≥4.5 years	<4.5 years	*χ* ^2^ *p* value^c^
Total (*n*)		392 (100)	195	197	
Sociodemographic factors					
Gender	Male	122 (31.1)	67	55	NS
	Female	270	176	128	
Age (years)	≥45	127 (32.4)	70	57	NS
	≥36 to <45	148 (37.8)	75	73	
	18 to <36	117	50	67	
Education	College or above	68 (17.3)	35	33	NS
	Secondary school	132 (33.7)	65	67	
	Primary or below	192	95	97	
Income (KES^d^)	≥10,000	79 (20.1)	41	38	<0.05
	≥5,000 to <10,000	119 (30.4)	47	72	
	<5,000	194	107	87	
Marital status	Currently married	166 (42.3)	77	89	NS
	Previously married	163 (41.6)	84	79	
	Never married	63	34	29	
Religion	Protestant	196 (50.0)	88	108	<0.05
	Catholic	138 (35.2)	68	70	
	Other	58	39	19	
Occupation	Paid employment	197 (50.3)	91	106	NS
	Homemaker	119 (30.4)	64	55	
	None	76	40	36	
Clinical factors					
Clinical symptoms	≥1 symptom	209 (53.3)	109	100	NS
	No symptoms	183	86	87	
Chronic illnesses	≥1 chronic illness	115 (29.3)	76	39	<0.05
	No chronic illness	277	119	158	
WHO stage at start	Stage 4	63 (16.1)	54	9	<0.05
	Stage 3	111 (28.3)	58	53	
	Stage 2	121 (30.9)	54	67	
	Stage 1	97	29	68	
Therapy-related factors					
Adherence^e^	Optimal	57 (14.5)	19	38	<0.05
	Fair	258 (65.8)	141	117	
	Poor	77	35	42	
ADRs	≥1 ADR	185 (47.2)	122	63	<0.05
	No ADR	207	73	134	
Therapy interruptions	≥1 interruption	253 (64.5)	156	97	<0.05
	No interruption	139	39	100	

ART: antiretroviral therapy; NS: not significant; WHO: World Health Organization; ADRs: Adverse drug reactions.

a Median duration on ART (4.5 years) used as cutoff for short versus long.

b Proportion displayed for each variable's levels but one.

c Pearson's chi-square test of independence.

d Kenyan shillings (USD 1.00≈KES 84.85 as of 4 July 2013).

e Refers to the multidimensional adherence explained in Annex I.

#### Clinical factors

Patients’ pretreatment clinical status was assessed using the WHO HIV stage at therapy initiation obtained from clinical records. Information on the patients’ current health was obtained from questions about their experience of acute and HIV-related constitutional symptoms in the 4 weeks preceding the interview as well as comorbidities. Checklists from prior studies assessing constitutional symptoms (17) and chronic illness burden in PLHIV (18) were used together with disease patterns data in the study area to generate responses for the questions on current chronic illnesses and symptoms of acute illness in the month prior to the study.

#### Therapy-related factors

Pharmacy and clinical records were used to obtain information on medication adherence, therapy interruptions, and clinic appointments. Medication adherence was weighted with the extent to which patients made clinic appointments on time. The algorithm is summarized in Annex I. The broader definition of adherence incorporates a behavioral aspect of HIV treatment that is consistent with the more comprehensive WHO definition of adherence (19) and builds on conceptual frameworks incorporating retention in care as a factor in defining engagement in HIV care (20).

### Statistical analyses

Sample characteristics were assessed using measures of central tendency and dispersion. Further analysis of dependence involved the Pearson chi-square test of independence and the OR.

In order to study the effect of duration on ART on HRQoL and how this effect improved the ability to predict membership in the levels of the binary HRQoL outcome variables after controlling for the potential confounders, a three-step hierarchical binomial regression analysis approach was used. In the initial step, all sociodemographic factors were entered into the regression equation, followed by the clinical factors. Clinical factors were included in the second step. In the final step, all of the therapy-related factors, including duration on ART, were added, and the changes in the predictive abilities of the models at each step were assessed using the likelihood ratio test and model accuracy. An additional step, where the duration variable was added separately from the other therapy-related factors, was used to establish the overall importance of the variable independent of the other theoretically related variables.

A two-tailed *p*-value of less than 0.05 was considered statistically significant for all hypothesis tests. Data were analyzed using Stata/SE 12.0 and IBM^®^ SPSS^®^ Statistics.

## Results

### Sample characteristics

A total of 392 patients participated in the study, representing a 93% response rate. Table 1 summarizes the patient characteristics and their associations with the primary independent variable. ORs (not shown) were computed for each variable in the table, with the last level being the reference category. The sample was predominantly female (68.9%), and the overall mean age was 41.4 (*SD*=8.3) years. The final gender distribution was consistent with the gender distribution in the sampling frame (69.0% female) and target population (69.8% female). Except for religion and income, other sociodemographic factors were not significantly associated with duration on ART, indicating that duration was independent of the participants' age, gender, education level, marital status, and occupation. Table 1 also shows that the participants more likely to report a preexisting chronic condition were also those who had been longer on ART (OR: 2.6, 95% CI [confidence interval]: 1.64–4.07). Adherence was associated with duration on ART (with adherence<80% as the reference; 80%≤adherence<95% OR: 3.9, 95% CI: 2.29–6.56; and adherence≥95% OR: 12.8, 95% CI: 5.99–27.47).

The median time on ART for the sample was 4.5 years (interquartile range: 2.6–6.5 years), and this was used to dichotomize the duration on ART variable for the regression analyses.

### Predicting HRQoL in PLHIV

#### Mental component scores

In the baseline model (Table 2), age (OR: 0.7, 95% CI: 0.55–0.97), gender (OR: 2.1, 95% CI: 1.31–3.44), religion (OR: 1.8, 95% CI: 1.35–2.48), and occupation (OR: 1.5, 95% CI: 1.16–2.05) were significant predictors of MCS in the absence of clinical and therapy-related factors. In step 2, age ceased to be a significant predictor, whereas gender's effect depreciated by 14%, indicating that the two were fully and partially statistically mediated by one or more clinical factors, respectively. Addition of therapy-related factors failed to improve the model (LR *χ* ^2^=8.2, not significant (NS)), and none of the therapy-related factors were significant.

**Table 2:** Hierarchical binomial logistic regression analysis of the MCS of the SF-36 against sociodemographic, clinical, and therapy-related factors showing odds ratios and 95% CI (n=392)

Independent variable^a^	Step 1^b^	Step 2^c^	Step 3^d^
Age (years)	0.7 (0.55–0.97)	0.8 (0.61–1.10)	0.9 (0.64–1.19)
≥45	0.5 (0.30–0.98)	0.7 (0.36–1.27)	0.8 (0.41–1.51)
≥36 to <45	0.6 (0.32–0.97)	0.6 (0.36–1.14)	0.7 (0.39–1.28)
Sex	2.1 (1.31–3.44)	1.8 (1.09–3.00)	1.9 (1.12–3.13)
Education	1.0 (0.73–1.33)	0.9 (0.67–1.25)	0.9 (0.69–1.30)
College or above	0.8 (0.44–1.61)	0.7 (0.38–1.47)	0.8 (0.39–1.54)
High school	1.2 (0.73–1.94)	1.1 (0.66–1.82)	1.1 (0.66–1.85)
Monthly income (KES)	1.1 (0.79–1.40)	1.1 (0.78–1.42)	1.0 (0.74–1.38)
≥10,000	1.0 (0.55–1.81)	1.0 (0.54–1.89)	0.9 (0.48–1.82)
≥5,000–10,000	1.5 (0.93–2.55)	1.5 (0.87–2.51)	1.4 (0.78–2.33)
Marital status	1.1 (0.81–1.46)	1.1 (0.81–1.49)	1.0 (0.76–1.42)
Currently married	1.1 (0.56–1.99)	1.1 (0.55–2.08)	1.0 (0.51–1.98)
Previously married	0.8 (0.45–1.59)	0.9 (0.45–1.64)	0.8 (0.43–1.64)
Religion	1.8 (1.35–2.48)	1.9 (1.36–2.55)	1.8 (1.33–2.52)
Protestant	3.5 (1.82–6.92)	3.7 (1.85–7.38)	3.6 (1.77–7.23)
Catholic	2.2 (1.11–4.33)	2.3 (1.15–4.73)	2.3 (1.14–4.79)
Occupation	1.5 (1.16–2.05)	1.5 (1.10–1.98)	1.5 (1.11–2.02)
Paid employment	2.5 (1.36–4.58)	2.3 (1.23–4.33)	2.4 (1.24–4.45)
Homemaker	1.5 (0.77–2.93)	1.6 (0.77–3.12)	1.5 (0.75–3.07)
Clinical symptoms		0.5 (0.32–0.78)	0.5 (0.32–0.78)
Chronic illnesses		0.4 (0.25–0.68)	0.4 (0.26–0.73)
WHO stage at start		1.0 (0.83–1.28)	1.1 (0.86–1.36)
Stage 4		1.0 (0.51–2.13)	1.2 (0.53–2.54)
Stage 3		1.1 (0.61–2.10)	1.2 (0.61–2.23)
Stage 2		0.9 (0.50–1.67)	0.9 (0.49–1.70)
Adherence			0.8 (0.55–1.19)
Optimal			0.6 (0.28–1.44)
Fair			0.9 (0.53–1.72)
Adverse drug reactions			0.7 (0.43–1.15)
Therapy-interruptions			1.5 (0.87–2.47)
Duration on ART (years)			0.8 (0.56–1.08)
≥5			0.6 (0.30–1.19)
≥3.5 to <5			0.7 (0.35–1.42)
Likelihood ratio test	*χ* ^2^≥41.7, *p*<0.05^e^	*χ* ^2^≥26.7, *p*<0.05^f^	*χ* ^2^≥8.2, *NS* f
Accuracy (%)	63.5	68.1	69.6

Note: Both sigma-coded and overparameterized models used. MCS: mental component score; ART: antiretroviral therapy; CI: confidence interval; KES: Kenyan shillings; NS: not significant; WHO: World Health Organization.

a Reference category for variable indicated in Table 1.

b Regression step with sociodemographic factors only.

c Regression step with sociodemographic and clinical factors only.

d Final model with all of the variables.

e Comparison with the constant-only model (null deviance).

f Comparison with the previous step's model.

#### Physical component scores

The three-step hierarchical regression analysis yielded incrementally preferred models from the baseline to the final model, both with respect to deviance (from LR *χ* ^2^=48.4, *p*<0.05, to LR *χ* ^2^=15.5, *p*<0.05) and to model accuracy (from 63.0 to 70.2%) (Table 3). In the baseline model, all sociodemographic factors except gender and income were significant PCS predictors. In step 2, clinical factors fully explained the effect of age. In the final model, duration on ART was a significant predictor (OR: 0.7, 95%: CI 0.47–0.93), with a distinct downward-sloping effect size gradient (duration 3–5 years, OR: 0.8, 95% CI: 0.41–1.65; and duration ≥5 years, OR: 0.5, 95% CI: 0.24–0.99). The presence of clinical symptoms and chronic illnesses was predictive of poorer PCS (OR: 0.5, 95% CI: 0.34–0.87; and OR: 0.3, 95% CI: 0.19–0.56, respectively).

**Table 3:** Hierarchical binomial logistic regression analysis of the PCS of SF-36 against sociodemographic, clinical, and therapy-related factors showing odds ratios and 95% CI (n=392)

Independent variable^a^	Step 1^b^	Step 2^c^	Step 3^d^
Age (years)	0.7 (0.51–0.90)	0.8 (0.59–1.07)	0.9 (0.65–1.21)
≥45	0.5 (0.28–0.92)	0.7 (0.36–1.29)	0.8 (0.42–1.62)
≥36 to <45	0.6 (0.32–0.98)	0.7 (0.38–1.22)	0.8 (0.42–1.41)
Sex	1.5 (0.93–2.43)	1.2 (0.75–2.07)	1.4 (0.82–2.32)
Education	1.4 (1.07–1.97)	1.4 (1.02–1.94)	1.5 (1.08–2.10)
College or above	1.9 (1.00–3.72)	1.9 (0.92–3.77)	2.2 (1.07–4.55)
High school	1.6 (0.96–2.52)	1.5 (0.88–2.42)	1.7 (1.00–2.89)
Monthly income (KES)	0.9 (0.69–1.23)	0.9 (0.68–1.26)	0.8 (0.60–1.14)
≥10,000	0.8 (0.43–1.42)	0.8 (0.41–1.50)	0.6 (0.32–1.21)
≥5,000–10,000	1.3 (0.79–2.19)	1.3 (0.76–2.25)	1.1 (0.63–1.94)
Marital status	0.7 (0.51–0.95)	0.7 (0.49–0.93)	0.6 (0.45–0.88)
Currently married	0.4 (0.22–0.82)	0.4 (0.19–0.78)	0.3 (1.16–0.68)
Previously married	0.5 (0.27–0.98)	0.5 (0.25–0.97)	0.5 (0.22–0.92)
Religion	1.9 (0.36–2.52)	1.9 (1.36–2.60)	1.8 (1.31–2.55)
Protestant	3.3 (1.69–6.43)	3.4 (1.67–6.74)	3.1 (1.49–6.34)
Catholic	1.7 (0.85–3.33)	1.7 (0.82–3.40)	1.4 (0.69–3.02)
Occupation	1.5 (0.15–2.06)	1.5 (1.08–1.98)	1.5 (1.10–2.03)
Paid employment	2.3 (1.22–4.16)	2.0 (1.06–3.87)	2.1 (1.09–4.10)
Homemaker	1.2 (0.61–2.34)	1.2 (0.59–2.47)	1.3 (0.61–2.66)
Clinical symptoms		0.6 (0.35–0.88)	0.5 (0.34–0.87)
Chronic illnesses		0.3 (1.17–0.48)	0.3 (0.19–0.56)
WHO stage at start		1.0 (0.82–1.28)	1.2 (0.93–1.53)
Stage 4		1.1 (0.51–2.23)	1.8 (0.79–4.13)
Stage 3		1.1 (0.60–2.08)	1.4 (0.70–2.68)
Stage 2		0.9 (0.48–1.64)	1.0 (0.51–1.83)
Adherence			0.7 (0.46–1.03)
Optimal			0.4 (1.19–1.05)
Fair			0.4 (0.24–0.82)
Adverse drug reactions			0.8 (0.51–1.38)
Therapy-interruptions			0.8 (0.47–1.35)
Duration on ART (years)			0.7 (0.47–0.93)
≥5			0.5 (0.24–0.99)
≥3.5 to <5			0.8 (0.41–1.65)
Likelihood ratio test	*χ* ^2^≥48.4, *p*<0.05^e^	*χ* ^2^≥35.2, *p*<0.05^f^	*χ* ^2^≥15.5, *p*<0.05^f^
Accuracy (%)	63.0	68.4	70.2

Note: Both sigma-coded and overparameterized models used. PCS: physical component score; ART: antiretroviral therapy; CI: confidence interval; KES: Kenyan shillings; WHO: World Health Organization.

a Reference category for variable indicated in Table 1.

b Regression step with sociodemographic factors only.

c Regression step with sociodemographic and clinical factors only.

d Final model with all of the variables.

e Comparison with the constant-only model.

f Comparison with the previous step's model.

#### HRQoL scores (aggregated PCS and MCS)

Table 3 shows that the predictive utility of the models, from the baseline to the final, improved in both deviance (from LR *χ* ^2^=39.1, *p*<0.05, to LR *χ*_2_=11.5, *p*<0.05) and accuracy (from 69.6 to 73.2%). Results from steps 1 and 2 were practically comparable, save for a noticeable 16% reduction in gender's effect size in step 2. In the final step, duration on ART was a significant predictor of HRQoL score, as was adherence (OR: 0.6, 95% CI: 0.45–0.92; and OR: 0.6, 95% CI: 0.41–0.96, respectively). Similar to the PCS regression, a negative dose-dependent-like effect size gradient was discerned for the association between duration and HRQoL scores. The effect of age was fully statistically mediated in the final model, suggesting that the variance in classification of overall health scores explained by age was indistinguishable from that explained by the therapy-related factors.

#### Sensitivity analyses

In order to test whether duration on ART significantly changed the models’ predictive ability independently, the variable was removed from the cluster of therapy-related variables and added in a fourth step separately. Results (not shown) indicated that duration remained significant in the fourth model, and it performed better than the step 3 model (without duration; HRQoL scores step 4: LR *χ* ^2^=6.0, *p*<0.05; PCS model step 4: LR *χ* ^2^=5.7, *p*<0.05; and MCS model step 4: LR *χ* ^2^=2.3, NS).

The multidimensional adherence variable was disaggregated, and the individual medication adherence and visit adherence variables were entered separately as part of the therapy-related factors into the final step. The two did not significantly alter other variables’ CIs or model fit, and they were not significant predictors in any of the three final models. It was thus inferred that they did not contribute much to the models on their own relative to the combined variable.

## Discussion

The main finding in this observational cross-sectional study is that duration on ART was found to predict poorer overall self-perceived health status after controlling for sociodemographic, clinical, and other therapy-related factors. From the analysis of sociodemographic factors, better HRQoL was associated with male gender, Christian faith, higher education, and paid employment, although the exact mechanism through which they (particularly gender and age) impact health status may be partially statistically explained by aspects of therapy and current clinical status (Tables 2, 3, and 4).

**Table 4:** Hierarchical binomial logistic regression analysis of the aggregated MCS and PCS of SF-36 against sociodemographic, clinical, and therapy-related factors showing odds ratios and 95% CI (n=392)

Independent variable^a^	Step 1^b^	Step 2^c^	Step 3^d^
Age (years)	0.6 (0.46–0.84)	0.7 (0.52–0.98)	0.8 (0.54–1.05)
≥45	0.4 (0.22–0.78)	0.5 (0.27–1.03)	0.6 (0.29–1.18)
≥36 to <45	0.4 (0.25–0.78)	0.5 (0.26–0.89)	0.5 (0.27–0.96)
Sex	2.1 (1.27–3.44)	1.7 (1.03–2.94)	1.9 (1.12–3.28)
Education	1.1 (0.77–1.44)	1.0 (0.70–1.36)	1.0 (0.72–1.43)
College or above	1.0 (0.48–1.91)	0.8 (0.36–1.60)	0.8 (0.38–1.74)
High school	1.3 (0.78–2.17)	1.2 (0.72–2.10)	1.3 (0.73–2.21)
Monthly income (KES)	0.9 (0.64–1.78)	0.9 (0.62–1.19)	0.8 (0.56–1.11)
≥10,000	0.7 (0.34–1.26)	0.7 (0.33–1.36)	0.6 (0.27–1.19)
≥5,000–10,000	1.4 (0.83–2.38)	1.3 (0.75–2.30)	1.2 (0.65–2.10)
Marital status	0.8 (0.60–1.13)	0.8 (0.58–1.13)	0.8 (0.53–1.06)
Currently married	0.6 (0.30–1.16)	0.6 (0.28–1.17)	0.5 (0.24–1.04)
Previously married	0.7 (0.34–1.30)	0.7 (0.30–1.32)	0.6 (0.29–1.24)
Religion	1.8 (1.28–2.53)	1.8 (1.27–2.57)	1.8 (1.22–2.51)
Protestant	3.3 (1.51–7.15)	3.3 (1.46–7.26)	3.0 (1.30–6.72)
Catholic	1.8 (0.83–4.11)	1.9 (0.82–4.29)	1.8 (0.75–4.10)
Occupation	1.7 (1.21–2.26)	1.6 (1.15–2.21)	1.6 (1.15–2.24)
Paid employment	2.7 (1.37–5.29)	2.6 (1.28–5.37)	2.7 (1.29–5.53)
Homemaker	1.3 (0.62–2.88)	1.4 (0.60–3.10)	1.4 (0.59–3.11)
Clinical symptoms		0.5 (0.30–0.78)	0.5 (0.30–0.79)
Chronic illnesses		0.3 (0.14–0.52)	0.3 (0.16–0.59)
WHO stage at start		1.1 (0.86–1.36)	1.2 (0.91–1.52)
Stage 4		1.2 (0.53–2.61)	1.5 (0.63–3.62)
Stage 3		1.7 (0.88–3.28)	1.8 (0.87–3.56)
Stage 2		1.2 (0.62–2.33)	1.2 (0.58–2.31)
Adherence			0.6 (0.41–0.96)
Optimal			0.4 (0.14–0.90)
Fair			0.6 (0.34–1.20)
Adverse drug reactions			1.0 (0.58–1.70)
Therapy-interruptions			1.4 (0.83–2.53)
Duration on ART (years)			0.6 (0.45–0.92)
≥5			0.4 (0.21–0.93)
≥3.5 to <5			0.8 (0.37–1.55)
Likelihood ratio test	*χ* ^2^≥39.1, *p*<0.05^e^	*χ* ^2^≥32.3, *p*<0.05^f^	*χ* ^2^≥11.5, *p*<0.05^f^
Accuracy (%)	69.6	72.7	73.2

Note: Both sigma-coded and overparameterized models used. MCS: mental component score; PCS: physical component score; ART: antiretroviral therapy; CI: confidence interval; KES: Kenyan shillings; WHO: World Health Organization.

a Reference category for variable indicated in Table 1.

b Regression step with sociodemographic factors only.

c Regression step with sociodemographic and clinical factors only.

d Final model with all of the variables.

e Likelihood ratio test for comparison with the constant-only model.

f Comparison with the previous step's model.

### Duration on ART

Although this study assessed patients on ART over a relatively wider time span than most prior work (9, 10, 21), the results are largely consistent with findings from similarly designed studies. Tran (22) present results that show a decline or stagnation in mean subscale scores after 4 years on treatment in five of the six HRQoL health domains measured, although the instrument used was different, thereby limiting direct comparisons. The mechanism through which duration on ART influences HRQoL has not been extensively studied and is therefore unclear.

Based on the notion of treatment fatigue in HIV treatment (23, 24), however, we posit that, with time, HRQoL becomes sensitive to cumulative adverse drug effects and personal and professional adjustments to one's life. This may in part explain the short-term HRQoL gains commonly observed in the 12-month longitudinal studies. This proposition is strengthened by studies (25) that suggest that in Kenya, labor participation is significantly lower in PLHIV than in the general population. A related postulation is that, as the clinical condition of PLHIV asymptotically approaches normalcy, the engagement between patients and healthcare providers may wane. On the health system's part, this may happen when the health system's capacity is not matched with increasing treatment coverage, thereby shifting clinical focus to the newly enrolled patients or those in need of immediate attention. Complacency on the patients’ part contributes to poorer medication adherence, dissatisfaction, and noncompliance with clinicopharmaceutical instructions (26), all of which may translate to conceivably poorer HRQoL in the long run.

### Pre- and posttreatment clinical status

Participants who had concurrent chronic comorbidities and/or had experienced clinical symptoms of an acute illness in the previous month were more likely to rate their health states significantly lower than those who had been free of the same, irrespective of duration on ART or other therapy-related factors. This would appear reasonable, since such physiological aberrations typically manifest physically, emotionally, and socially, hence affecting the perception of one's general health (5, 27).

In the analysis, duration on ART was associated with both chronic comorbidities and pretreatment WHO HIV stage (Table 1). This may imply a greater disease burden (both clinical and self-assessed) among those on ART for longer periods. This presents a policy opportunity to mitigate possibly temporally worsening health status in PLHIV. It is with this in mind that investments in routine screening of patients for constitutional clinical symptoms and other health problems, irrespective of treatment stage or with greater focus on those on ART for longer duration, should be made so that psychosocial and other complications associated with low HRQoL may be averted.

### The paradox of adherence

In this study, a multidimensional measure of adherence was used (Annex I), justified by an *a priori* understanding of adherence in HIV and the *post-hoc* sensitivity analyses. Adherence was negatively associated with HRQoL, independent of duration on ART, and the negative association strengthened away from the null with increasing adherence. While most studies have found high adherence to be a significant predictor of better HRQoL (28, 29), others have found no association (30). Though this may be design related, since this study captured both phenomena simultaneously thereby resulting in the observation that better adherers were also at a poorer health state and vice versa, two possibilities arise. Firstly, *because* of poorer pretreatment or deteriorating in-treatment health status, patients may be more motivated (both internally and externally) to increase their adherence to medication and instructions (multidimensional adherence). Conversely, as a patient's health approaches normalcy relative to their baseline status *because* of treatment, they may relax their compliance to medication and other instructions (26).

### Limitations

This being a cross-sectional study, causal relationships cannot be conclusively determined. Nonetheless, cross-sectional findings prompt hypotheses justifying further investigations – a case in point here being the puzzle of adherence. Though the study sought to control for known potential confounders, omissions are expected and acknowledged – the most prominent being psychosocial factors such as stigma, social support networks, depression, disclosure, and occupation-related health. We cannot rule out, based on current literature (31, 32), their possibly independent impacts on HRQoL in PLHIV, and further research into the interplay between such factors and those used in this study should yield greater understanding of the issue at hand.

In addition, since participant selection was partly based on clinic attendance, those with appointments outside the study period may have been missed. Post-hoc cross-tabulation showed no significant differences between the two groups with respect to gender and age, but those not selected were more likely to have been on ART for longer. This is expected as they usually have widely spaced clinic appointments.

## Conclusion

Patients’ assessment of their well-being during HIV treatment was negatively associated with their duration on treatment irrespective of sociodemographic, clinical, and other therapy-related variables. This may have implications in the design and implementation of effective interventions to improve the uptake and retention of patients in HIV treatment programs that take a treatment-stage-targeted approach. In light of the strong negative association between constitutional symptoms and chronic comorbidities with both treatment duration and HRQoL, HIV care programs may benefit from strategies that strengthen the patient–provider relationship with a particular focus on those on treatment for a relatively longer duration. Finally, further research into the role of a wider definition of adherence in HIV research is recommended.

## Annex I

Medication adherence was calculated from manual pill count data using the formula:

$$A_{d\cdot m\cdot f}=\frac{\sum_{x=1}^{X}P_{d-d\cdot m\cdot f}-PC_{d\cdot m\cdot f}}{R_{f}\times days\left(d,d-X\right)}$$

where *A*_*d·m·f*_ is the adherence computed for the *m* ^*th's*^ participant's *f* ^*th*^ medicine dispensed on the *d* ^*th*^ visit; *R*_*f*_ is the *f* ^*th*^ medicine's dosing information (number of pills per day); *PC*_*d·m·f*_ is the pill count for the *f* ^*th*^ medicine presented by the *m* ^*th*^ participant on their *d* ^*th*^ visit; *X* is the number of visits before the *d* ^*th*^ visit for which adherence is quantifiable; the summation of P_*d* −*x·m·f*_ for all *X* is the total number of pills dispensed to the participant for the *X* visits; and days is a function computing the difference (in days) between the *d* ^*th*^ visit and *X* visits before that. The numerator is therefore the actual number of pills consumed during the period *d*−*X*, and the denominator is the total number of pills expected to be consumed during the same period. Note that this is a specialization of the widely used Medication Possession Ratio (MPR) that takes into account within-course therapy regimen changes and deferred pill counts. The participants’ cumulative medication adherence was then computed as the arithmetic mean for all medicines and for all of the visits. For categorical analysis, the medication adherence variable was transformed into a trinomial variable taking one of the following values: ‘optimal’ (medication adherence ≥0.95), ‘moderate’ (0.95>medication adherence≥0.80), and ‘low’ (medication adherence<0.80).

Clinic appointment compliance was obtained as the number of visits on time (give or take 5 days) divided by the total number of visits. For categorical analyses, the variable was transformed into a trinomial variable taking one of the following values: ‘optimal’ (appointment compliance ≥0.90), ‘moderate’ (0.75>appointment compliance≥0.90), and ‘low’ (appointment compliance<0.80).

To create the multidimensional adherence variable, medication adherence and appointment compliance were combined with the *AND* and *OR* Boolean logical operators to produce three levels as follows: ‘optimal’ (medication adherence ≥0.90 *AND* appointment compliance ≥0.80), ‘fair’ (medication adherence ≥0.85 *AND* appointment compliance<0.80) *OR* (appointment compliance ≥0.75 *AND* medication adherence<0.90), and ‘low’ (medication adherence<0.85 *AND* appointment compliance<0.75).
